# *In Vitro* Pre-validation of Gene Editing by CRISPR/Cas9 Ribonucleoprotein

**Published:** 2019

**Authors:** Maryam Mehravar, Abolfazl Shirazi, Mohammad Mehdi Mehrazar, Mahboobeh Nazari

**Affiliations:** 1. Reproductive Biotechnology Research Center, Avicenna Research Institute, ACECR, Tehran, Iran; 2. Monoclonal Antibody Research Center, Avicenna Research Institute, ACECR, Tehran, Iran; 3. Research Institute of Animal Embryo Technology, Shahrekord University, Shahrekord, Iran

**Keywords:** CRISPR/Cas9, In vitro digestion, Ribonucleoprotein

## Abstract

**Background:**

The CRISPR/Cas9 genome editing system is a powerful and simple gene editing method. The format of the CRISPR components is one of the important factors in targeting efficiency. Compared to plasmid or mRNA (IVTs) format, using the CRISPR/Cas9 system as Cas9–crRNA–tracrRNA RNP format is more efficient and rapid, especially in minimizing some of the pitfalls of CRISPR-mediated gene editing. In addition to efficient *in vivo* applications of the CRISPR RNP format in a variety of cell types and organisms, another advantage of this approach is usability for *in vitro* applications in which the crRNAs in the tracrRNA–crRNA structure guides the Mg2+-dependent RNAdirected DNA endonuclease to introduce double-strand breaks at specific sites in DNA.

**Methods:**

Here, Cas9–crRNA–tracrRNA RNP system was used to test the designed crRNAs for in vitro DNA cleavage by Cas9 protein in RAG1, RAG2 and IL2RG genes.

**Results:**

The results of cleavage reveal theCas9–crRNA–tracrRNA RNP system is a rapid and efficient way to pre-validate the efficiency of CRISPR cleavage with crRNAs designed for RAG1, RAG2 and IL2RG genes.

**Conclusion:**

one step *in vitro* cleavage of DNA by CRISPR/Cas9 ribonucleoprotein complex can be used to pre-validate the functionality and relative efficiency of CRISPR system for targeting genes.

## Introduction

Targeted genome editing through site-specific endonucleases have been developed in recent years. Genome editing technologies based on endonucleases like Zinc Finger Nuclease (ZFN) and Transcription Activator–Like Effector Nuclease (TALEN) have been used for targeted genome modifications 1–3. These proteins are rather complex to design, need to be assembled for each target sequence 4,5 and the process for protein engineering can be complicated and time consuming.

Recently, the Clustered Regularly Interspaced Short Palindromic Repeats (CRISPR) system associated to the Cas9 endonuclease (CRISPR/Cas9) has been developed as a specific and effective tool for genome engineering. It is inexpensive and easy to carry out 6–8.

CRISPR/Cas9 system consists of a *Streptococcus pyogenes (S. pyogenes*) derived Cas9 nuclease and a RNA duplex made of a CRISPR RNA (crRNA) and a trans-activating crRNA (tracrRNA) 9 that would be replaced by a synthetic single guide RNA (sgRNA) chimera that mimics the crRNA: tracrRNA duplex 10.

This system is based on the base-pairing of the DNA sequence, adjacent to an obligate Protospacer Adjacent Motif (PAM) NGG, with a short complimentary RNA sequence which is then cleaved by the Cas9 protein sequence-specific manner 8,11,12. Double Strand Breaks (DSBs) induced by this system can be repaired in one of the two ways: Non-Homologous End Joining (NHEJ) or Homology-Directed Repair (HDR) and introducing two sgRNAs along with Cas9 may result in deletions 13.

Cas9 is a large protein with two nuclease domains, RuvC-like domain near the N-terminus and a HNH (His-Asn-His)-like domain is in the middle of the protein. The Cas9 HNH domain cleaves the complementary DNA strand, while the RuvC-like domain cleaves the non-complementary DNA strand 10,14.

The crRNA consists of the 16–22 nucleotides deriving from 3’ end of the repeat sequence and 20 nucleotides complementary to the DNA strand of protospacer (target site), which guides Cas9 to the DNA target 14. The tracrRNA is partially complementary to crRNA and required for crRNA maturation and DNA cleavage by Cas9 9,10,14. For specific DNA cleavage by Cas9 protein, two conditions are required; firstly, a short nucleotide sequence 5′-NGG-3′, called a PAM (Protospacer-adjacent motif) that is located in the vicinity of a protospacer and secondly, binding to the target site (a protospacer) complementary to the crRNA. DNA cleavage in protospacer introduced by the Cas9 protein of *S. pyogenes* occurs 3 nt away from the PAM sequence 10,14. The RNA-guided Cas9 nuclease from *S. pyogenes* (Cas9sp) is widely used to introduce *in vivo* genetic alterations in a variety of cells and organisms including bacteria, yeast, monkeys and human cell lines 15.

CRISPR/Cas9 can be introduced as DNA plasmid, In Vitro Transcriptions (IVTs) or as Ribonucleoprotein (RNP) format in which the recombinant Cas9 protein is assembled *in vitro* with chemically synthetized sgRNA or crRNA: tracrRNA duplex. Guide RNAs generated by IVT or chemical synthesis for RNP system completely bypass cloning process and allow generation and validation of a CRISPR reagent faster. Also, above formats except RNP can not be applicable *in vitro* and require to be delivered into cultured cells that involve time-consuming cell culture and transfection steps 16.

CRISPR/Cas9 system as a programmable molecular tool can be widely used for in vitro applications 17,18.

In previous methods, crRNA and tracrRNA have been oftenly used as single guide RNA (sgRNA) that were artificially made by humans in which a linker was added between the two crRNA and tracrRNA pieces to make them one single RNA. Recently, in RNP complex, a group of scientists have designed a new approach to use CRISPR, called the innovative Alt-R CRISPR-Cas9 System that is based on the natural *S.* pyogenes CRISPR RNA system and utilizes the optimized crRNAs and tracrRNAs that were shortened to 36 and 67 nucleotides, respectively. This system improves the potential of genome editing, saves time by utilizing ready-to-use RNA reagents, and reduces cell toxicity by avoiding activation of cellular innate immune responses (www.idtdna.com/CRISPR-Cas9).

RNPs are the most rapid, transient, convenient and efficient format of using *CRISPR/Cas9* gene editing 19. Also, by using this method, researchers could be able to minimize some of the pitfalls of CRISPR-mediated gene editing such as mosaicism and off-target effects 20–22.

Cas9 RNP complex is not limited by the amount and rate of Cas9 translation, and pre-loaded sgRNAs are likely to be protected from degradation 22. In addition to efficient *in vivo* applications of CRISPR RNP, this method can be applicable for the *in vitro* applications unlike the former plasmid-based approach and IVT method. Therefore, to pre-validate CRISPR/Cas9 RNP complex for genome editing in various organisms, there is no need to test the system first in respective cell lines and only an *in vitro* cleavage assay is enough for this purpose.

As the plasmid-based approach for CRISPR-mediated genome editing of *RAG1* and *RAG2* genes in the mouse Embryonic Stem Cells (mESCs) and mouse zygote was used in our previous work, first, an attempt was made to test the system in the NIH3T3 cell lines (Mouse embryonic fibroblast cells). This process is time-consuming and requires preparation of steps such as cell culture, transfection, selection, DNA isolation, PCR and T7 endonuclease1 assay for the cleavage analysis.

In order to test the functionality and relative efficiency of CRISPR RNP system, *in vitro* cleavage of DNA was carried out in one step by CRISPR/Cas9 ribonucleoprotein complex.

**Figure 1.**
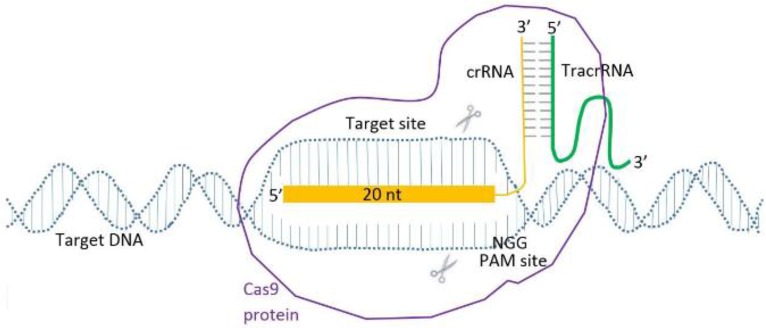
Alt-RcrRNA: *tracrRNACas9 RNP gene* targeting.

The crRNAs that show activity were selected for CRISPR-mediated gene targeting of the mouse ESCs and zygote. Obligatory components of Cas9 RNP system were provided to cleave specific target sites of *RAG1*, *RAG2* and *IL2RG* genes that are involved in immune system functions. The components included the Mg^2+^-dependent RNA-directed DNA endonuclease (Recombinant Cas9 protein), crRNA with a specific sequence complementary to the protospacer, tracrRNA, the fragment of each gene with a specific protospacer sequence and a PAM (Figure 1).

## Materials and Methods

### Target selection

The 20-nt target sites that are complementary to the 5′ end of the crRNA on each gene were selected using online CRISPR RGEN Tools program (https://www.rgenome.net) based on specificity rules, including uniqueness of the selected sites throughout the genome, 5′-G-N_19_-NGG-3′ matching and selection of target sequences in coding region nearby gene promoter, because target sites near the 5′ end of Coding DNA Sequence (CDS) are more efficient as they produce early frame shifts and stop codons 23.

### In vitro assembly of a Cas9 ribonucleoprotein complex

To assemble CRISPR RNP complex, duplex RNA (crRNA: tracrRNA) was prepared by annealing crRNA to tracrRNA. For annealing, 5 *μg* of crRNA (5 *μl* of 1 *μg/μl*) and 10 *μg* of tracrRNA (10 *μl* of 1 *μg/μl*) for each gene were mixed and annealed in a thermocycler by heating to 95*°C* for 5 *min* and cooling to 25*°C*. To form RNP complex, an incubation step is required either before *in vitro* cleavage assay or during incubation time of cleavage assay. In this experiment, RNP complexes were formed in one step as described in the following section. But for the experiments of CRISPR-mediated gene targeting in cell lines or zygote, the RNP complex was first formed *in vitro* and then delivered into cells.

### DNA cleavage by in vitro assembled Cas9 RNP complex

The DNA cleavage activity was assayed on PCR products of *RAG1*, *RAG2* and *IL2RG* genes that had been amplified by a pair of PCR primers for each gene (Table 1) and containing target site and PAM sequences. As mentioned, RNP complexes were formed during cleavage reaction by mixing crRNA: tracrRNA with Alt-R *S. pyogenes* Cas9 Nuclease 3NLS activated in 10X Cas9 nuclease reaction buffer (Nacl 1 *M*, MgCl_2_ 0.1 *M*, tris-HCL 0.5 *M*, BSA 1 *mg/ml*, pH= 7.9). All components were ordered from Integrated DNA Technologies (IDT).

**Table 1.** Primers used to amplify fragments of *RAG*1, *RAG2* and *IL2RG* genes

**Primer names**	**Sequence 5′ to 3′**
*RAG1*-primer F	GAAGAAGCACAGAAGGAGAAG
*RAG1*-primer R	ATCGGCAAGAGGGACAATAGC
*RAG2*-primer F	ATTCCTCCTGGCAAGACT
*RAG2*-primer R	GCATACACTCTGACAAGCA
*IL2RG*-primer F	TGACACAGACTACACCCAGAG
*IL2RG*-primer R	TCAGCCCTTTAGACACACCAC

The cleavage reaction (30 µl) was performed by mixing the following components: 1 µl duplex RNA (1 μg/μl), 3 µl 10X Cas9 nuclease reaction buffer, 3 µl Cas9 enzyme (200 ng/µl), ddH_2_O (To final volume 29 µl) and 1 µl respective PCR product for each gene (100 nM). Then, the mixtures were incubated at 37°C for 2 hr. The reactions without adding duplex RNA were considered negative controls for each duplex RNA. After incubation, 1 μl Proteinase K (20 mg/ml) was added to the reaction and then the mixture incubated at 65°C for 10 min to release the DNA from the Cas9 endonuclease. When both crRNAs were active on RAG2 and IL2RG genes, to test the activity of both crRNAs, the corresponding reaction was performed. The products of each reaction were assessed by electrophoresis on 2% agarose gel. The results of cleaving RAG1 and RAG2 genes were compared to the results of our previous work when plasmid-based CRISPR-mediated gene targeting was used.

## Results

### crRNA selection

Target sequences that are complementary to the 5′ end of the crRNAs were designed based on rules mentioned in materials and methods. Finally, target sequences in coding regions of *RAG1*, *RAG2* and *IL2RG* genes were selected so that one sequence was targeted in *RAG1* gene and two sequences were targeted in each of *RAG2* and *IL2RG* genes. According to target selection, the crRNAs were ordered from IDT (www.idtdna.com/CRISPR-Cas9) in their proprietary Alt-R format (Table 2).

**Table 2.** crRNA sequences

**SgRNA names**	**Sequence 5' to 3'**
*RAG1*	CGCGAGACGGGACCGTCGCA
*RAG2-1*	GAATGGCCGTATCTGGGTTC
*RAG2-2*	TGCTTTTCCCTCGACTATAC
*IL2RG-1*	ATCTGATAATAATACATTCC
*IL2RG2*	TTCTGTACAGCTCGCCTCTG

### Targeted cleavage of RAG1, RAG2, IL2RG genes in vitro

**Figure 2.**
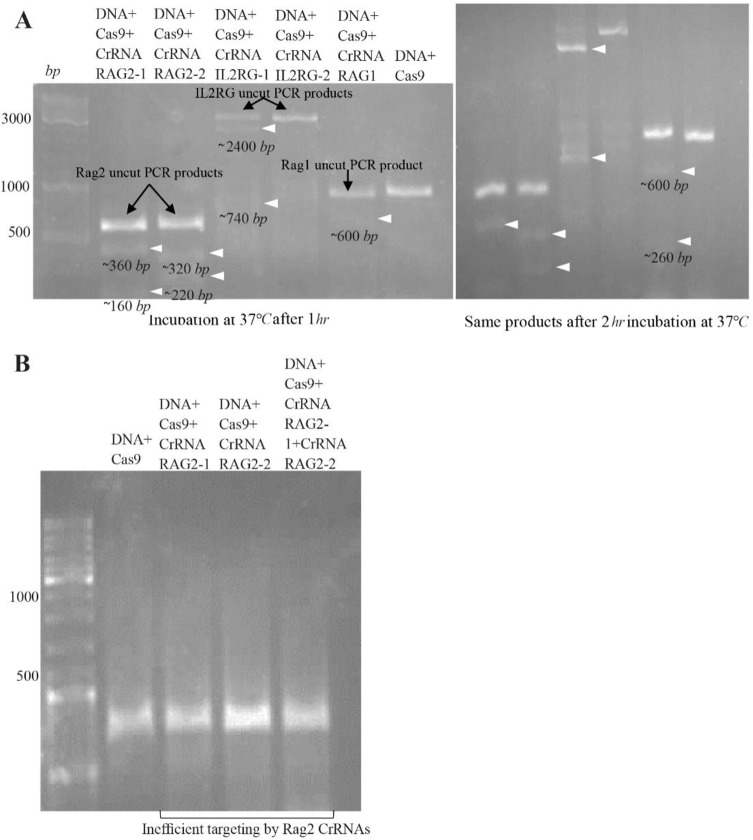
*In vitro* DNA cleavage using Cas9 nuclease and crRNAs (CRISPR RNP). A) Cas9 nuclease and crRNAs for targeting *RAG1*, *RAG2* and *IL2RG* genes. Left: incubation at 37*°C* after 1 *hr*. Lane1, 2: first bands are the Rag2 uncut PCR products, second and third bands are cleaved fragments represented by white arrows. Lane 3, 4: first bands are the IL2RG uncut PCR products, second and third bands are cleaved fragments represented by white arrows. Lane 5: first band is the Rag1 uncut PCR product, second band is one of the cleaved fragments, another cleaved fragment of Rag1 disappeared on agarose gel electrophoresis but after 2 *hr*, a faint band was found. Lane 6: Rag1 PCR product as a DNA control. Right: incubation at 37*°C* after 2 *hr*. The lanes are in the same order just after 2 *hr*. B) Cas9 nuclease and crRNAs for targeting *RAG2* genes after 6 months of shelf life. Very faint bands show inefficient targeting by Rag2 crRNAs. In all agarose gel pictures, bands resolution is not brilliant due to low quality of GelRed.

The *in vitro* digestion of three genes was analyzed by agarose gel electrophoresis. The results show that crRNA RAG1, crRNA RAG2-1 and crRNA RAG2-2 are active and crRNA-guided Cas9 specifically cleaves target DNA sequences of *RAG1* and *RAG2* gene fragments. Only one crRNA IL2RG (IL2RG-1) had activity, thus crRNA IL2RG-2 was not appropriate to use for *CRISPR* gene targeting of mouse genome (Figure 2A). The results of *in vitro* digestion of *RAG1* and *RAG2* gene fragments by Cas9 guided by crRNA RAG1, crRNA RAG2-1 and crRNA RAG2-2 were consistent with the results of plasmid-based CRISPR gene targeting of NIH3T3 cells for RAG1 and RAG2 sgRNA in our previous work (submitted to AJMB). But compared to the plasmid-based method, it can be used very simply and quickly, so that the approximate duration of the plasmid method lasts about two weeks, while in this method the whole procedure can be done in one day.

## Discussion

Therefore, *in vitro* digestion of targeted genes by *CRISPR/Cas9* gene editing in RNP format can be a simple and rapid method for pre-validation of CRISPR/Cas9 system before using in the cell types and organisms without the need to deliver CRISPR components into cells. But the point that may not be a drawback for RNP complex is that the shelf life of the chemically synthetic crRNA and tracrRNA in the best condition is 6 months and after this time CRISPR components do not work efficiently; likewise, this system was used after 6 months and the results showed the system did not work efficiently (Figure 2B).

## Conclusion

In conclusion, one step *in vitro* cleavage of DNA by CRISPR/Cas9 ribonucleoprotein complex can be used to pre-validate the functionality and relative efficiency of CRISPR system for targeting RAG1, RAG2 and IL2RG mouse genes. Although the shelf life of the RNP components is limited but to test the validation of CRISPR/Cas9 system, plasmid-based approach compared to the RNP system is much more time consuming and laborious.
